# Characterizing thermal tolerance in the invasive yellow-legged hornet (*Vespa velutina nigrithorax*): The first step toward a green control method

**DOI:** 10.1371/journal.pone.0239742

**Published:** 2020-10-06

**Authors:** Ignacio Ruiz-Cristi, Laurence Berville, Eric Darrouzet

**Affiliations:** IRBI, UMR 7261 CNRS—University of Tours, Tours, France; Wildlife Conservation Society Canada, CANADA

## Abstract

The yellow-legged hornet, *Vespa velutina nigrithorax* (Hymenoptera: *Vespidae*, Lepeletier 1836), is native to Southeast Asia and has been unintentionally introduced in France. The species is spreading in many areas of the world. The European Union has classified *V*. *velutina* as a species of concern because the hornet significantly affects beekeeping activities, mostly by preying honeybees (*Apis mellifera*) at beehive entrances. No current control method is simultaneously eco-friendly and effective. Here, we aimed to develop a greener technique for destroying *V*. *velutina* nests, inspired by a defense behavior used by the eastern honeybee (*Apis cerana*), the “heat ball”. In the laboratory, we tested how *V*. *velutina* of different sexes, castes, and developmental stages responded to different heat exposure systems employing a range of temperature levels. Overall, the time of death decreased as temperature increased. Hornets died faster when the temperature was gradually increased than when it was instantaneously increased; larvae seemed to be more thermally tolerant. The most promising and potential technique for quickly destroying hornet nests may be steam injection, as the humid airflow system killed all hornets within 13 seconds, and therefore could be a good candidate for a green nest control method.

## Introduction

Invasive species are among the leading threats to native wildlife [1–3], and their presence requires ever more intensive management approaches. A species is considered to be invasive when it spread over a large area outside their native range, producing negative impacts on biodiversity, human health, and activities [1, 4, 5]. Human travel and international trade are largely responsible for spreading non-native species, frequently across the entire globe, and often unintentionally [6]. Invasive species pose a direct threat by outcompeting native species for food or other resources [7], preying on native species, and causing or carrying disease [8–11]. The introduction of a predator into a new ecosystem can have disastrous consequences for economic activities [12, 13]. Once invasive species have become established, they are extremely expensive to eradicate [14, 15], and successful eradication remains rare [16, 17]. The yellow-legged hornet, *Vespa velutina nigrithorax* (Hymenoptera: Vespidae), is native to tropical and subtropical regions of Southeast Asia [18]. Around 2004, it was unintentionally introduced into southwestern France from China [19]. This eusocial species has rapidly spread across most of continental France and into other parts of Europe [20, 21]. It now occurs in Spain, Portugal, Italy, Germany, Belgium, the Netherlands, and the United Kingdom [21]. It is also found in South Korea [22] and Japan [23, 24].

Hornets’ nests are founded by a single queen and developed annually; colonies can produce thousands of individuals and thus require a steady supply of proteins and carbohydrates to function [25]. To feed the larvae, adult hornets prey on a wide variety of arthropods [25, 26]; in Europe, their main food source is the honey bee (*Apis mellifera*) [27, 28]. Hornets capture foraging honey bees at beehive entrances during the critical pre-wintering season (September and October) [29, 30], leading to a decrease in honey production in the following year, which has major effects on beekeeping activities [27]. As a result, the hornet’s impact on honey bee colonies can extend well beyond direct predation. The presence of *V*. *velutina* is associated with several serious issues (loss of biodiversity, reduced economic activity, and threats to human health, including death [27, 31], which led the European Union (EU) to include it in the list of invasive alien species of Union concern [32]. While physical and biological control methods have been developed [33], none are simultaneously eco-friendly and effective. Most are baited traps that use proteins or sugars [33], which generally kill many non-target insects and few *V*. *velutina* [34, 35]. Consequently, indiscriminate mass trapping has significant environmental impacts on the local entomofauna [36] but does not appreciably drive down *V*. *velutina* populations. Another control method is to locate and destroy entire nests. However, at present, there is no eco-friendly way to efficiently do so. The best current approach is to inject nests with permethrin [33], a biocide with a half-life of 28–38 days on the ground, and 10 days on vegetation [37–39]. However, because of the nest’s protective envelope, the compound could potentially remain active for even longer inside the nest. As a result, non-target organisms, like insects and birds, could also be affected [40].

To elaborate an eco-friendly method for destroying *V*. *velutina* nests, we looked to natural systems. In particular, two biological phenomena could be mimicked. First, western honey bees (*Apis mellifera*) can kill Oriental hornets (*Vespa orientalis*) via asphyxia-balling: honey bees mob hornets and suffocate them [41]. Second, eastern honey bees (*Apis cerana*) have an effective collective defense mechanism for countering the hornet’s attacks. When a hornet approaches the nest, a group of bees rush out and surround it, forming a “heat ball.” The temperature inside the ball is around 47°C, which is above the lethal temperature limit of the hornet (45.7°C) but not for the bees [42–45]. The bees can survive at temperatures up to 50.7°C [46]. This mechanism has been found to be effective against *V*. *simillima* [42], *V*. *velutina* [44], *V*. *magnifica* [47], and *V*. *multimaculata* [48].

It could be interesting to mimick, in the future, this defense mechanism observed in *A*. *cerana*. However, before to use heat to kill a hornet colony, it is necessary to select first the temperature to use and how to increase heat in the nest. In this study, we thus explored whether yellow-legged hornets of different sexes, castes, and developmental stages could be killed using temperature-based control methods. We hypothesized that hornets will not be able to survive above 50°C and that temperature will be enough to kill the complete colony. It is the first step in determining whether such approaches could be used as alternatives to chemical compounds, which are harmful to the environment. More specifically, we used different heat exposure systems employing a range of temperature levels to assess overall and group-specific time of death of hornet and determine which heat exposure system might be best at instantly immobilizing hornets in the idea to destroy their colonies in the future.

## Materials and methods

### Collection and rearing of hornet colonies

**Fig 1:**
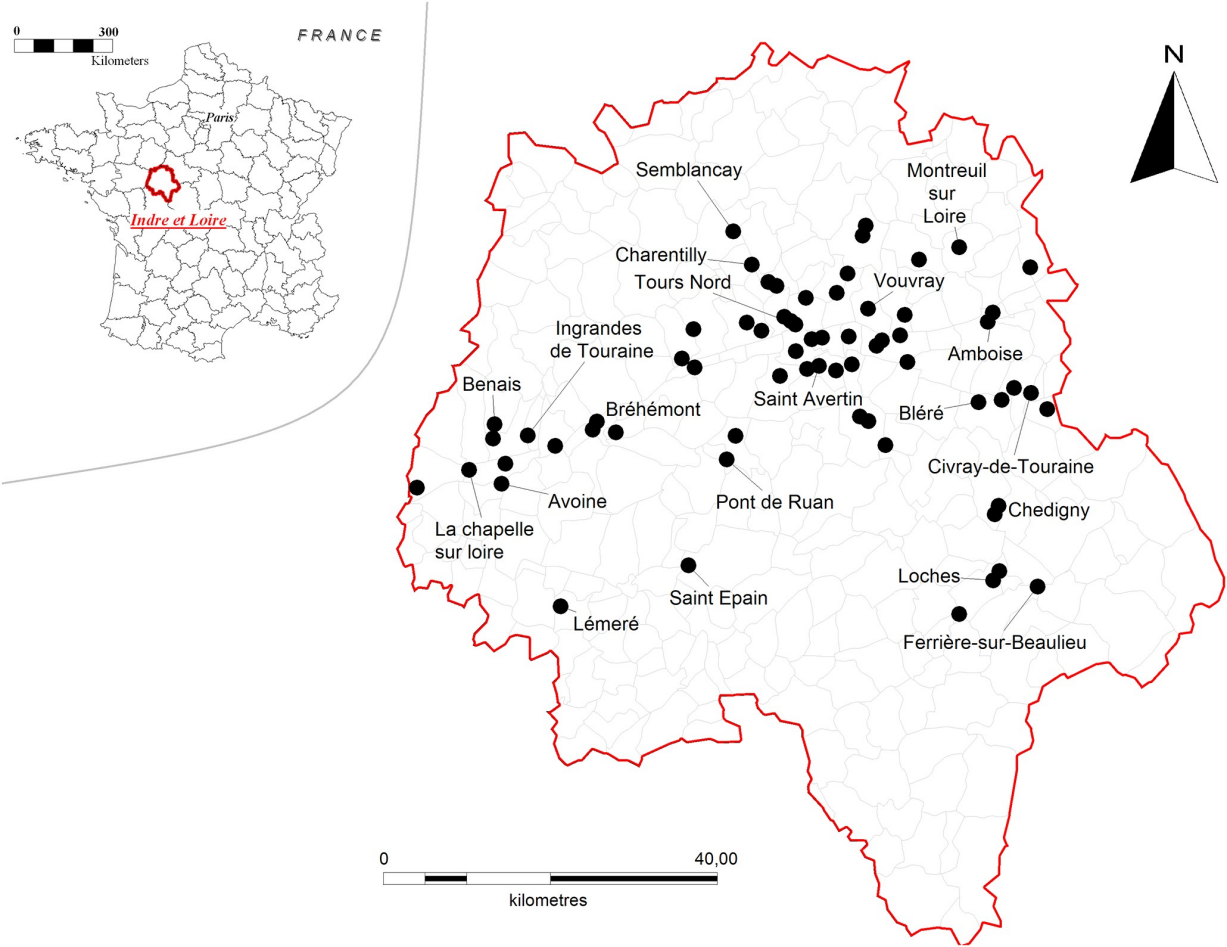
Location where yellow-legged hornets (*Vespa velutina nigrithorax*) colonies were sampled (Department of Indre-et-Loire, France).

Hornet colonies were collected from June to November 2019 (n = 66) in western central France (Department of Indre-et-Loire; Fig 1 and S1 Table). As the yellow-legged hornet is officially considered as an invasive species, no permits were required for insect collection, and all studies were carried out in compliance with relevant national guidelines. They were brought back to the laboratory and housed in separate boxes left at room temperature (19–22°C). To determine the identity of each female (worker vs. reproductive), individuals were weighed, and their wing spacing was measured [49]. All types of individuals (workers, gynes, males, and queens) were given daily a solution of water and honey *ad libitum*. On the day of colony collection, larvae were removed from the combs and used immediately in the experimental trials. The adults were used as quickly as possible after collection (on average 2.2 days, SD±: 3.2). Before each experimental trial, hornets were anesthetized using carbon dioxide (CO_2_). As soon as a hornet woke up, the trial began.

### Experimental trials

To test the individual thermal tolerance of yellow-legged hornets on the time of death, four heat exposure systems were assessed.

#### Instantaneous & gradual heat

First, we explored the use of instantaneously applied radiant heat (hereafter, the flash system; n = 31 colonies) and gradually applied radiant heat (hereafter, the gradual system; n = 50 colonies); four temperature levels were used (50°C, 60°C, 70°C, and 80°C). Both systems were applied to the colony as a whole: all sexes (males and females), castes (workers and reproductives), and developmental stages (larvae and adults). For both systems, we performed 30 replicates using gynes, males, workers, and larvae; across the replicates, on average 6 different colonies (SD± 2.07) were represented (n = 120 at each temperature level for each system). Due to the scarcity of queens, they were only used in these first two systems (at each temperature level, n = 5 for the flash system and n = 3 for the gradual system). Second, two direct airflow systems were used to evaluate the influence of humidity on the time of death of adult hornet: one system employed dry heat (hereafter, the dry airflow system), and the other system employed moist heat (hereafter, the humid airflow system). Three temperature levels were used (100°C, 120°C, and 140°C). The dry airflow system was tested using just workers (n = 90), while the humid airflow system was tested using gynes, males, and workers (n = 90). In all the experimental trials, the temperature was measured with a digital thermometer (HI 935005, ± 0.2°C). All the trials were filmed using a Panasonic camera (4K, HC-VX980) to better quantify the time of death.

For both the flash and gradual systems, a water bath was used (Fisherbrand^™^ Polystat^™^ Immersion Circulator [±0.05°C], Bioblock Scientific). In the flash system, hornets were individually placed in an octagon glass jar (95 x 87 mm) closed with a lid. The jar had been previously placed in a water bath of the target temperature (50°C, 60°C, 70°C, or 80°C). A probe was inserted into the jar to measure its internal temperature. In the gradual system, hornets were individually placed in a glass tube (90 x 24 mm) at room temperature that was closed with a foam lid. Then, when the water bath reached the target temperature (50°C, 60°C, 70°C, or 80°C), the glass tube was put into the water. In both systems, we noted the exact time and temperature at which the hornets died. For the gradual system, calibration curves were established (n = 3 for each temperature), allowing us to determine the internal temperature of the glass tubes at the time of death (S1 Fig). The experimental trial ended when the hornet could no longer move. In all experiments, dead hornets were then observed outside the experimental system tested for 15 minutes to confirm their death.

#### Dry and humid airflow

High temperatures are needed to quickly immobilize the hornets and thus prevent them from attacking or escaping. In the dry airflow and humid airflow systems, high temperatures were combined with airflow. For the dry airflow system, we carried out preliminary tests on hornet nest in the laboratory to assess the potential fire hazard associated with different temperature levels (120°C, 130°C, 140°C, and 150°C, n = 15 each). Hot air at the target temperature was applied to a fragment of nest comb for 4 minutes using a heat gun (Tacklife HGP72AC, 2000 W/230 V, 500 l/min; n = 15 for each temperature level). The presence or absence of combustion, defined as the appearance of smoke and/or a color change in the comb, was noted. Using this information, we were able to select experimental temperatures that did not pose a fire risk: 100°C, 120°C, and 140°C. These temperatures were tester on hornets from 11 colonies. In the dry airflow trials, a hornet was placed in a cylindrical metal cage (55 x 85 mm) that was located 10 cm away from the heat gun. The trial ended when the hornet could no longer move and died. For the humid airflow system, hornets, from 11 colonies, were placed in a rapid bottle warmer (NUK Thermo Rapid, 500 W, 220V); they were surrounded by wire mesh (50 x 70 mm) and kept 0.9 cm away from the water (also using wire mesh). To create steam (mean temperature: 92.2°C), 6 ml of water was added to the bottle warmer before the machine was switched on. The interior temperature was recorded using a digital thermometer, as mentioned above.

### Statistical analysis

All the statistical analyses were performed using R (v. 3.1.2, R Development Core Team, 2014). The figures were generated using R or SigmaPlot (v. 12.5). First, data normality was assessed using a Shapiro-Wilk test. Then, to analyze how each system affected the time of death of hornet, Kruskal-Wallis one-way analysis of variance (ANOVA) on ranks using the time-to-death data was used; posthoc pairwise comparisons were performed using the Tukey test and the Dunn test. To determine whether there was a relationship between hornet mass and time to death, the Pearson correlation (with normal distribution) and the Spearman correlation (without normal distribution) were used. To assess how well the logarithmic calibration curves obtained using the gradual system reflected internal temperature in the glass tube, we calculated the coefficient of determination (R^2^).

## Results

**Fig 2:**
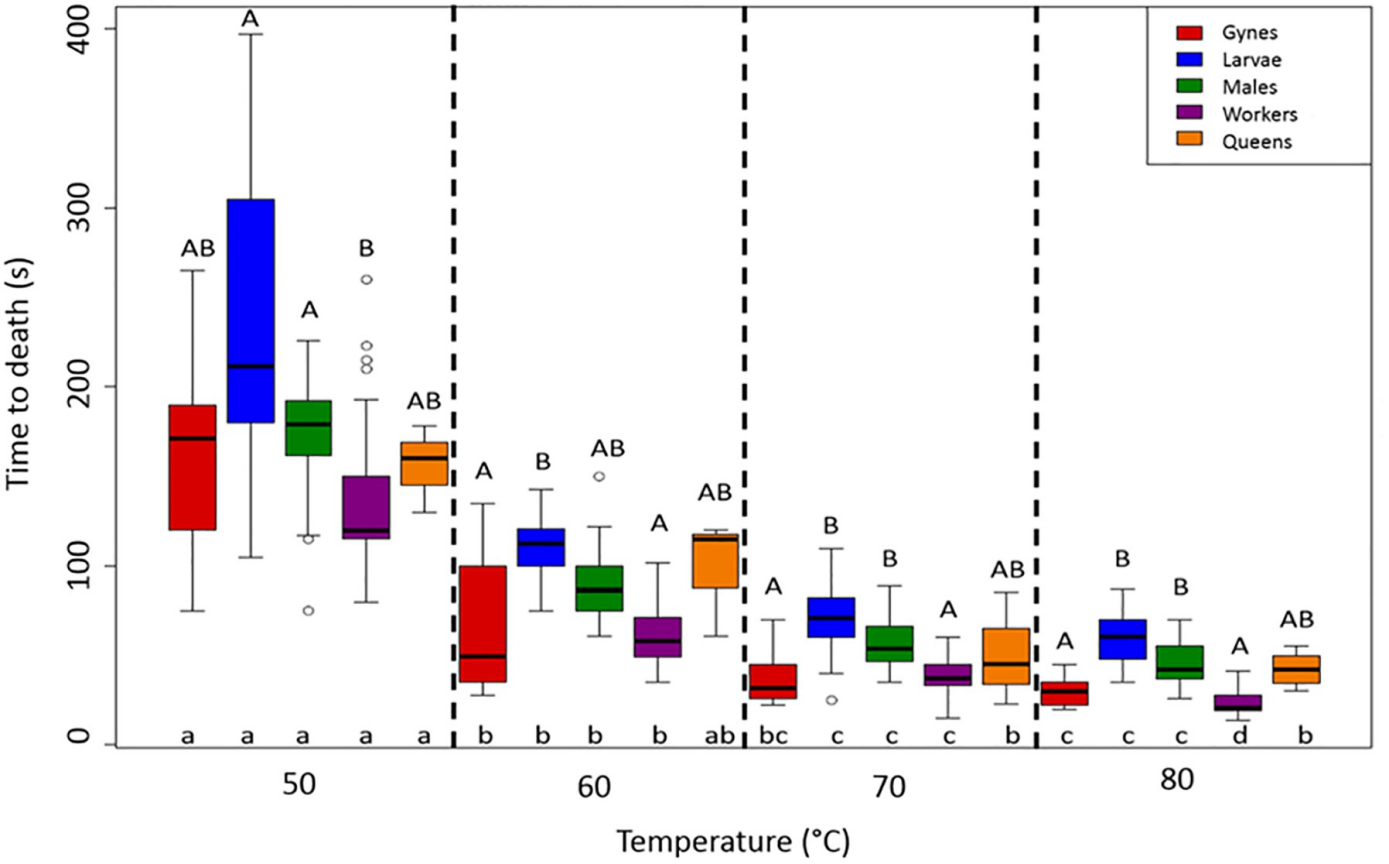
Effects of the flash heat exposure system on the time of death of gynes, larvae, males, and workers (n = 30 each) and queens (n = 5). Differences in the uppercase letters above the boxes indicate significant differences within a given temperature level (50°, 60°, 70°, and 80°C). Differences in the lowercase letters below the boxes indicate significant differences within each group (see the color legend) between temperature levels.

The individual thermal tolerance of all castes and life stages of the invasive hornet *V*. *velutina*, was assessed, through both an instantaneous and a gradual increase in temperatures. When the temperature was increased instantaneously (the flash system), all the hornets died within 7 minutes. Overall, hornets died significantly faster at higher temperatures (Kruskal-Wallis one-way ANOVA: H = 405.551, df = 19, P < 0.001); median time to death was 120 seconds (q_1_ = 115; q_3_ = 129) at 50°C, 56 seconds (q_1_ = 48; q_3_ = 69) at 60°C, 37 seconds at 70°C (q_1_ = 33; q_3_ = 44), and 21 seconds (q_1_ = 18; q_3_ = 29) at 80°C (Fig 2). There was a marked difference in the time of death of gyne between 50°C and the three higher temperatures (Tukey test: P < 0.05, q_50-60_ = 5.913, q_50-70_ = 9.387, _q50-80_ = 11.531) as well as between 60°C and 80°C (Tukey test: P < 0.05, q_60-80_ = 5.619). In contrast, no difference was seen between 60°C and 70°C or between 70°C and 80°C. Likewise, larvae died significantly more quickly as temperature increased (Tukey test: P < 0.05, q_50-60_ = 4.314, q_60-70_ = 5.999, q_50-70_ = 10.314, q_50-80_ = 12.140, q_60-80_ = 7.826; Fig 2), except between 70°C and 80°C. The same pattern was seen in males as in larvae (Tukey test: P < 0.05, q_50-60_ = 5.054, q_60-70_ = 5.154, q_50-70_ = 10.209, q_50-80_ = 12.859, q_60-80_ = 7.805). For workers, median time to death was 120 seconds at 50°C versus 21 seconds at 80°C (Tukey test: P < 0.05, q_50-60_ = 5.194, q_50-70_ = 9.437, q_50-80_ = 13.418, q_60-70_ = 4.244, q_60-80_ = 8.225, and q_70-80_ = 3.981). For queens, time to death was significantly different between 50°C and both 70°C and 80°C (Kruskal Wallis one-way ANOVA, Holm-Sidak method: t_50-80_ = 5.958, P = 0.001 and t_50-70_ = 5.145, P = 0.003, respectively; Fig 2); however, these results must be interpreted with caution because of the small sample sizes (n = 5 at each temperature level). Taking these results together, the larvae seemed to be the most thermally tolerant group, followed by the males. At 50°C, both males and larvae survived significantly longer than workers (Dunn test: P < 0.05, Q_males_ = 5.714, Q_larvae_ = 3.342); workers had similar time of death to gynes and queens. At 60°C, larvae survived significantly longer than both gynes and workers (Dunn test: P < 0.05, Q_gynes_ = 5.434, Q_workers_ = 6.473) but did not live any longer than males or queens. At 70°C and 80°C, larvae and males survived significantly longer than both workers and gynes (Dunn test: P < 0.05, Q_gynes-larvae70_ = 6.585, Q_gynes-larvae80_ = 6.523, Q_workers-larvae70_ = 6.065, Q_workers-larvae80_ = 8.134, Q_gynes-males70_ = 4.889, Q_gynes-males80_ = 3.791, Q_workers-males70_ = 4.369, Q_workers-males80_ = 5.403; Fig 2).

**Fig 3:**
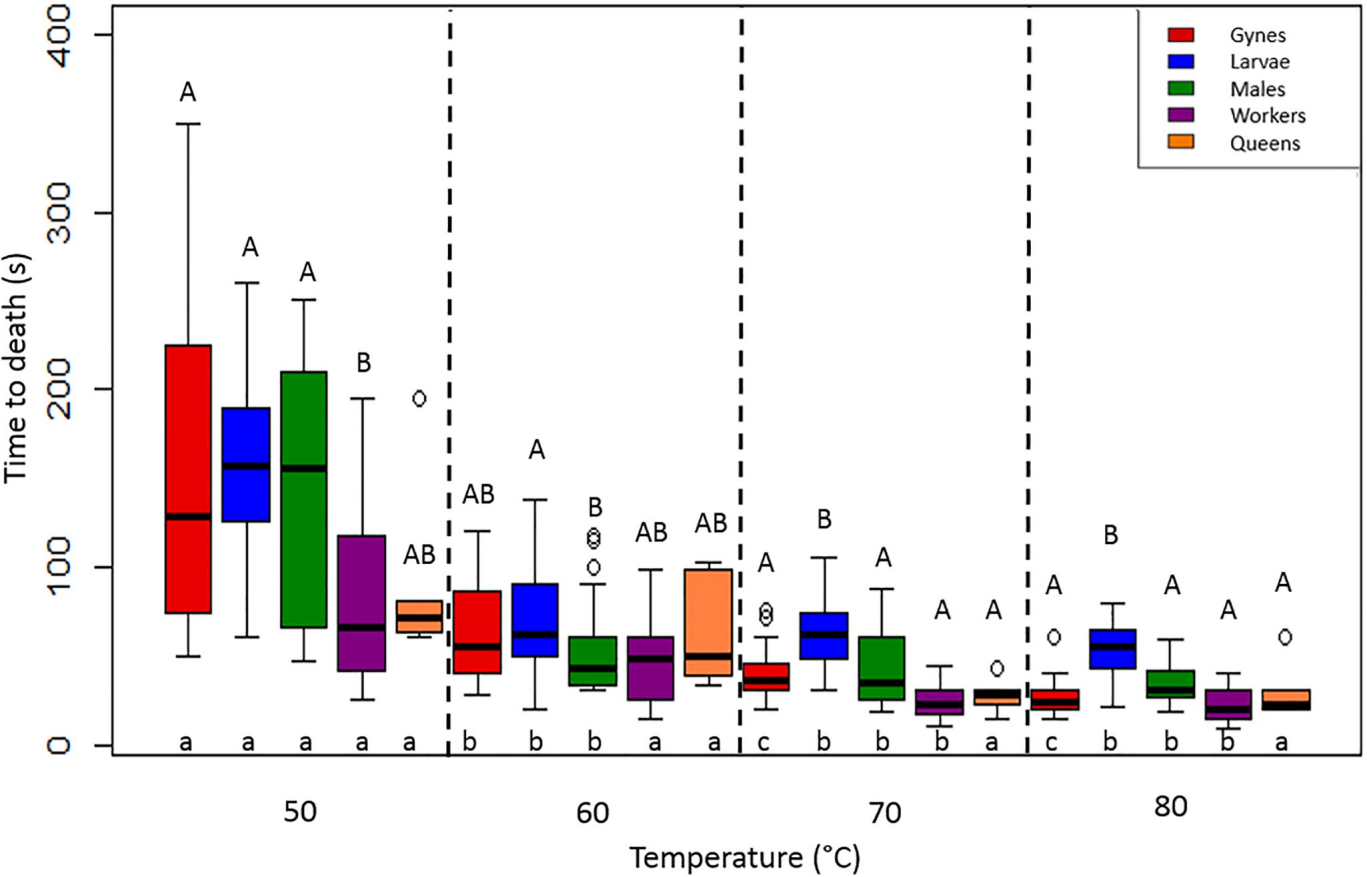
Effects of the gradual heat exposure system on the time of death of gynes, larvae, males, and workers (n = 30 each) and queens (n = 3). Differences in the uppercase letters above the boxes indicate significant differences within a given temperature level (50°, 60°, 70°, and 80°C). Differences in the lowercase letters below the boxes indicate significant differences within each group (see the color legend) between temperature levels.

When the temperature was increased gradually (the gradual system), all the hornets died within 6 minutes. Overall, hornets died significantly faster at higher temperatures (Kruskal-Wallis one-way ANOVA: H = 284.699, df = 19, P < 0.001; Fig 3): mean time to death was 125 (q1: 66; q3: 191) seconds at 50°C (45°C on the calibration curve), 52 (q1: 35; q3: 78) seconds at 60°C (48°C on the calibration curve), 38 (q1: 28; q3: 55.5) seconds at 70°C (53°C on the calibration curve), and 30 (q1: 20.5; q3: 43.5) seconds at 80°C (55°C on the calibration curve). In line with the overall results, gynes died significantly more quickly at higher temperatures, except between 70°C and 80°C (Tukey test: P < 0.05, q_50-60_ = 4.286, q_60-70_ = 3.992, q_50-70_ = 8.277, q_50-80_ = 11.392, q_60-80_ = 7.107; Fig 3). Larvae survived significantly longer at 50°C than at the three higher temperatures (Tukey test: P < 0.05, q_50-60_ = 7.298, q_50-70_ = 8.393, q_50-80_ = 10.091); no difference in time to death was observed between 60°C, 70°C, and 80°C. Males showed a similar pattern to the larvae (Tukey test: P < 0.05, q_50-60_ = 7.191, q_50-70_ = 7.592, q_50-80_ = 9.739; Fig 3), including the lack of difference in time of deathbetween 60°C, 70°C, and 80°C. Workers died significantly faster at the higher temperatures (70°C and 80°C) than at the lower temperatures (50°C and 60°C) (Tukey test: P < 0.05, q_50-70_ = 7.482, q_50-80_ = 9.652, q_60-70_ = 5.291, q_60-80_ = 7.461); no difference in time to death was observed between 70°C and 80°C. The queens displayed no difference in the time of death among temperature levels; however, these results must be interpreted with caution because of the small sample sizes (n = 3 at each temperature level) (Fig 3). Based on all the results, the larvae seemed to be the most thermally tolerant group. Indeed, at 50°C, workers died significantly faster than gynes, larvae, and males (Dunn test: P < 0.05, Q = 3.289, Q = 4.271, and Q = 3.050, respectively). At 60°C, males died significantly faster than larvae (Dunn test: P < 0.05, Q = 3.442). At 70°C and 80°C, larvae survived longer than any other group (Dunn test: P < 0.05, Q_workers70_ = 5.652, Q_gynes70_ = 3.747, Q_queens70_ = 3.139, Q_males70_ = 3.223, Q_workers80_ = 7.145, Q_gynes80_ = 5.609, Q_males80_ = 3.731; Fig 3).

System type had an effect: the gradual system killed hornets more quickly than did the flash system (Figs 2 and 3). Significant differences were observed for the workers at 50°C (Mann-Whitney U test: U = 208.5, P < 0.001), 60°C (U = 298.5, P = 0.025), and 70°C (U = 244.5, P = 0.002). The same was true for the males at all the temperature levels (50°C: U = 207, P < 0.001; 60°C: t-test, t = -8.086, P < 0.001; 70°C: U = 261.1, P = 0.005; and 80°C: U = 206, P < 0.001) and for the larvae at both 60°C and 80°C (60°C: U = 136.5, P < 0.001; 80°C: t = 2.016, P = 0.048; Figs 2 and 3).

**Fig 4:**
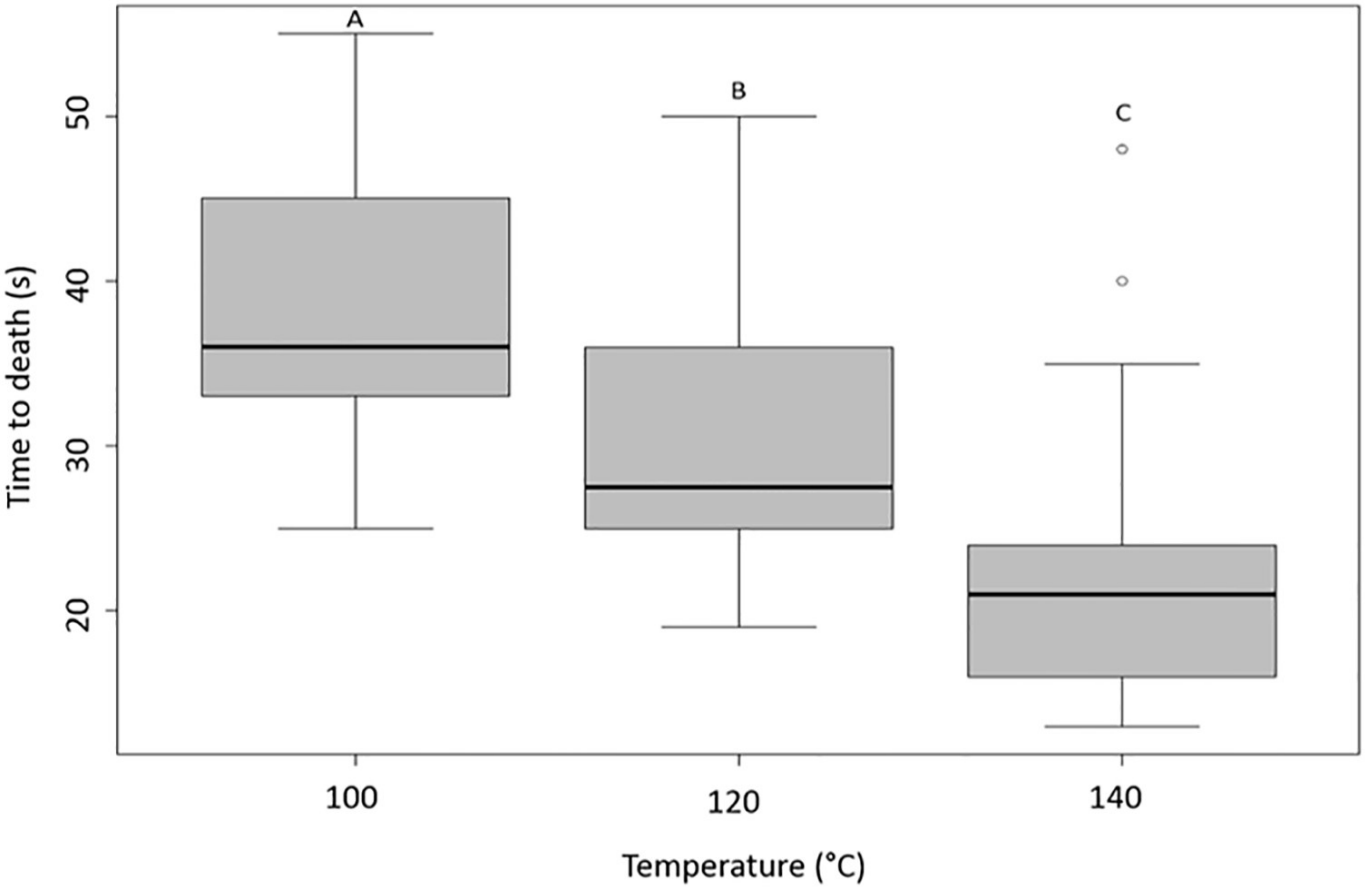
Effects of the dry airflow system on time of death of worker at 100°C, 120°C, and 140°C (n = 30 each). Differences in the letters above the boxes indicate a significant difference in time of death.

In the preliminary dry airflow trials, none of the combs showed signs of combustion at 120°C, 130°C, or 140°C (n = 15 at each temperature level). The combs exposed to 150°C did (n = 11 out of 15). Consequently, we only used the safe temperatures in the subsequent trials. Workers died more quickly at higher temperatures (Kruskal-Wallis one-way ANOVA: H = 39.772, df = 2, P < 0.001): mean time to death was significantly different between 100°C, 120°C, and 140°C (Tukey test: P < 0.05, q_140-100_ = 8.872, q_140-120_ = 5.144, q_100-120_ = 3.728; Fig 4). The median time to death for all workers was 36 (q1: 35.5; q3: 45) seconds at 100°C, 27.5 (q1: 24.75; q3: 36.25) seconds at 120°C, and 21 (q1: 16; q3: 24.5) seconds at 140°C (Fig 4).

**Fig 5:**
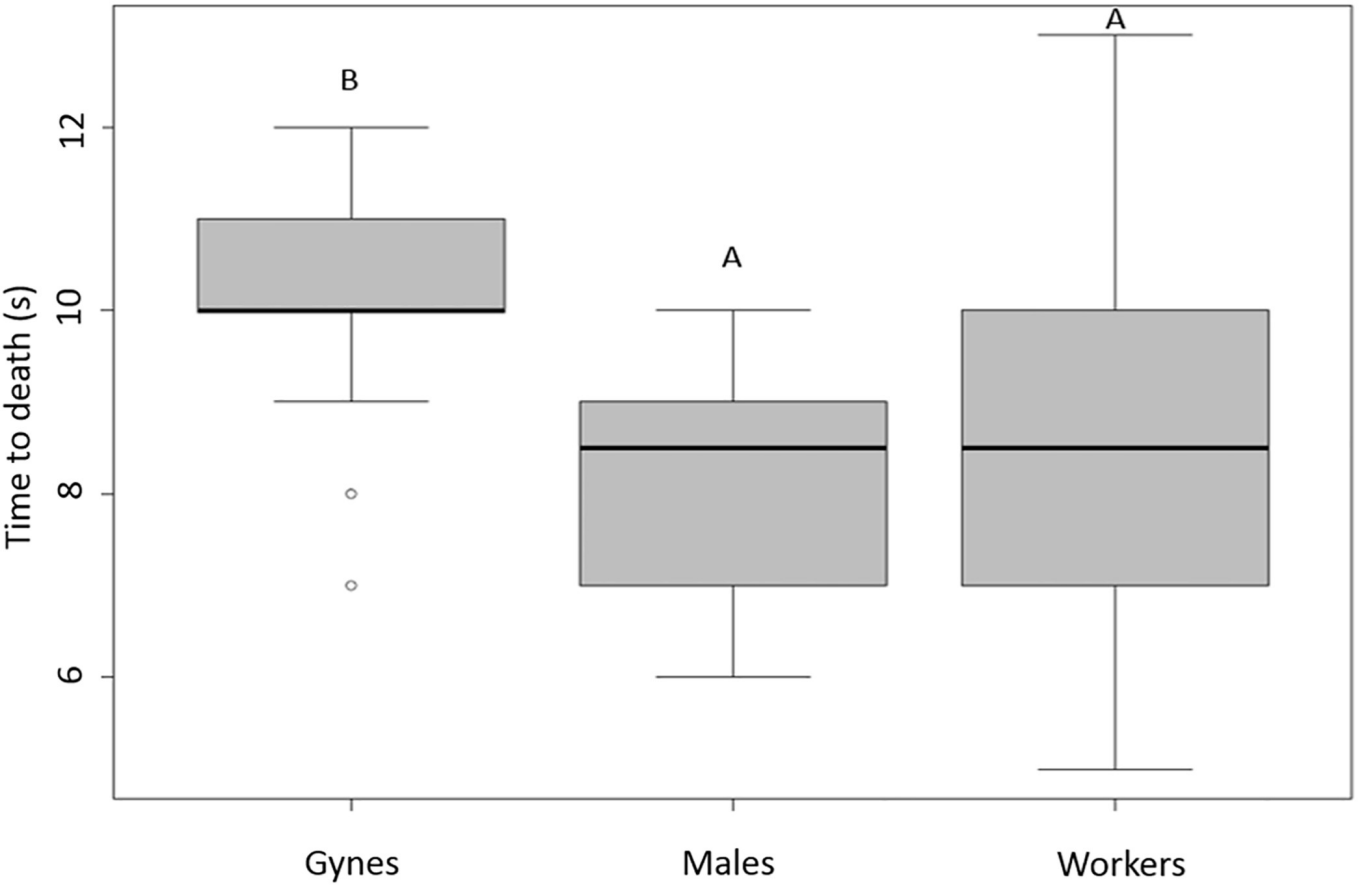
Effects of the humid airflow system on the time of death of gynes, males, and workers (n = 30 each). Differences in the letters above the boxes indicate a significant difference in time of death.

In the humid airflow trials, there were significant differences in time of death (Kruskal-Wallis one-way ANOVA: H = 30.573, df = 2, P < 0.001; Fig 5). Both males and workers died more quickly than gynes (Tukey test: P < 0.05, q_gynes-males_ = 7.432, q_gynes-workers_ = 5.388). The median time to death was 10 (q1: 10; q3: 11) seconds for gynes, 8.5 (q1: 7; q3: 9) seconds for males, and 8.5 (q1: 7; q3: 10) seconds for workers (Fig 5). The two airflow systems yielded different results for workers at 100°C (Kruskal-Wallis one-way ANOVA: H = 44.418, df = 1, P < 0.001; Figs 4 and 5): mean time to death was 38.2 seconds with dry airflow versus 8.5 seconds with humid airflow (Tukey test: q_vapor-Dry air (100°C)_ = 9.409, P < 0.001; Figs 4 and 5). There was no correlation between mass and time of death for workers, males, gynes, or larvae in 30 of the 38 systems tested (Pearson or Spearman correlations; S2 Table). Eight of them were significantly correlated (P < 0.05) but their correlation coefficients were close to 0.5; denoting no statistical relationship between the two variables.

## Discussion

Due to its negative impacts worldwide, the yellow-legged hornet (*V*. *v*. *nigrithorax*) is the target of various control efforts [33]. As there are no signs that the species’ expansion is slowing, it is essential to bolster detection and control strategies. In the future, the global economic costs of invasive species will increase rapidly [15, 50] and the effects of climate change on the invasive expansion will grow [50]. At present, current methods for controlling the yellow-legged hornet are limited: they are sometimes effective but not eco-friendly. Thus, new techniques must be developed.

Due to their small size and ectothermic physiology, insects are particularly vulnerable to temperature, which can have a direct effect on their behaviors (flight…), and fitness (lifespan…) [51]. For these reasons, the heat was previously tested in control methods against insect pests in different conditions, as in the museum [52] and food storage [53]. Classically, a true core temperature of above 45°C sustained for several hours will kill insect adults [53].

In this study, we found that humid airflow was the most efficient system for killing hornets (100% mortality within 13 seconds at 92.2°C), and it entailed no risk of nest combustion. In contrast, when dry airflow was used, workers survived for up to a minute at 100°C. Therefore, heat exposure alone does not suffice as a control method because it could give hornets time to flee. In natural condition, various species seem to use three complementary parameters for killing hornets: heat, CO_2_, and/or humidity. Cyprian honeybees (*A*. *m*. *cypria*) utilize heat-balls, which combine heat and CO_2_ to effectively smother Oriental hornets (*V*. *orientalis*) [41]. The same combined approach is used by Japanese honey bees (*A*. *c*. *japonica*) in their heat-balls [45] as they defend themselves against Japanese giant hornets (*V*. *mandarinia japonica*). Furthermore, Japanese and European honey bees can kill hornets (*V*. *mandarinia*, *V*. *analis*, *V*. *simillima*, and *V*. *crabro*) by forming heat-balls in which there is a simultaneous rapid increase in temperature, CO_2_, and humidity [54]. In the same study, it was shown that the giant hornet had a lower median lethal temperature at 90% relative humidity (under artificial laboratory conditions) than at 40–50% relative humidity (mean under average natural conditions). Many social insects can tolerate high temperatures (30°C to 60°C) thanks to evaporative cooling [55, 56]. Classical social insect behaviors for regulating nest temperature include directing warm air away from the nest (i.e., wing fanning) and evaporatively cooling the nest (i.e., workers collect water and place droplets on the surface of the brood comb, [57]). However, evaporative cooling, is no longer viable when humidity is high; consequently, temperature regulation is disrupted, and lower temperatures become more lethal [55, 56].

Here, according to our data obtained in the laboratory, the idea will be to quickly inject steam water into a nest, which should immobilize and kill hornets in few seconds. However, in the field, insects could escape the lethal thermal shock by taking refuge or fleeing. For this reason, it is crucial that any heat diffusion method be rapidly applied. Further studies are necessary to test the efficacy of our proposed control technique in the field on entire yellow-legged hornet colonies.

The data collected in these experiments also demonstrated that a gradual increase in temperature was more deadly to hornets than an instantaneous increase in temperature. Workers and males died significantly faster when the air temperature rose from room temperature to 50°C, 60°C, or 70°C. Indeed, more than 50% of the hornets had died before the target temperature was reached. For example, it took about 170 seconds for the temperature inside the glass tube in the 50°C water bath to climb from 21°C to 50°C (±0.1); however, most of the workers had died before then (93.3%). In contrast, when the temperature was instantaneously set at 50°C, only 70.6% of workers died. The median lethal temperature (44.6°C) was reached at around 134 seconds in the flash system versus at around 63 seconds in the gradual system. In an experiment using an incubator, where the temperature rose 1°C every 20 minutes from an initial setting of 42°C, it was observed that the yellow-legged hornet’s lethal thermal limit was 45±0.48°C [44], the hornets died after an average of 60 minutes in the incubator. In our study, in the gradual system, the temperature rose 1°C every 5 seconds until it reached 50°C. Previous research has shown that initial temperature and the rate of temperature change have a highly significant effect on critical thermal limits [58]. Here, it is possible that quickly and permanently increasing the temperature during extreme thermal stress wore out the hornet’s metabolism, preventing the organism from thermally acclimating or regulating its temperature. In contrast to what found with the tsetse fly (*Glossina pallidipes*) [58], we discovered that slower rates of temperature change (i.e., longer experimental trials) resulted in greater survival in the yellow-legged hornet. At 50°C, workers died significantly faster than did gynes, larvae, and males. This heat exposure system mimics the functioning of a heat-ball in honey bees (where a maximum temperature of 45.9 ± 1.0°C [45] is reached within 5–10 minutes [44]). Consequently, in nature, we can assume that it would take more time and effort for honey bees to kill a male or gyne yellow-legged hornet with a heat-ball. Of all the groups considered in this study, the larvae seemed to be the most thermally tolerant. However, because they cannot survive without the presence of adults, the larvae would die of starvation or become prey if all the workers died. Therefore, the temperature needed to kill the entire colony is the maximum temperature needed to kill the workers and the queen early in the season (in Europe: May to late July) and all the adults later on.

Thermal tolerance could be influenced by several natural factors that could not be controlled in this experiment. For example, hornets differ in their phenology and/or health status, and it might be that older workers are less resistant than younger workers. However, age does not appear to influence the critical thermal limits of insects [58]. In this study, there was variability in time to death at lower temperatures (50°C and 60°C) for workers and males. This variability was less pronounced at higher temperatures (70°C and 80°C). Overall, mass and time to death were not correlated, a result that contrasts with those of certain previous studies in social insects, in which a relationship between mass and thermal tolerance was observed. In the ant *Cataglyphis cursor*, large workers survived longer than small workers at 48°C and 52°C (application of radiant heat; [59]). It is possible that, in the yellow-legged hornet, the difference in mass between early-season hornets (around 350 mg) and end-of-season hornets (around 550 mg) is not large enough to evaluate the above relationship. Indeed, we did observe a trend of heavier workers surviving longer at 50°C, although the results were not significant. Another possibility is that the lowest temperature tested (50°C) was already quite high, given that the thermal tolerance of the hornet *V*. *velutina* is 45.9 ± 1.0°C [44]. Such a situation would leave no room for variation in phenology or health status to manifest itself.

In conclusion, we have shown that a method based on steam injection may hold the most promise for controlling the yellow-legged hornet given that humid airflow was capable of killing individual hornets within a few seconds. Such a technique would be eco-friendly, have limited collateral impacts, and pose no risk of combustion. The method’s speed will be crucial to its successful use in nature on entire nests. Future research must take place in the field to explore hornet behavior in response to steam injection, ascertain whether hornets have enough time to escape, and determine whether the steam acts upon the entire nest.

## Supporting information

S1 Table Geographic coordinates (World Geodetic System 1984, Decimal degrees) where yellow-legged hornets (*Vespa velutina nigrithorax*) colonies were sampled (Department of Indre-et-Loire, France). (PDF)

S2 Table Correlation between hornet mass and time to death for each method tested. Normality distribution was tested with Shapiro-Wilk method. Pearson correlation method (with normal distribution) and the Spearman correlation method (without normal disitribution) were used.
(PDF)

S1 Fig Calibration curves for the gradual heat exposure system. Color codes: gray = 50°C (R^2^ = 0.9), red = 60°C (R^2^ = 0.9), green = 70°C (R^2^ = 0.9), and yellow = 80°C (R^2^ = 0.9) (n = 3 for each).
(TIF)
