# Supplementary material for: Characterizing thermal tolerance in the invasive yellow-legged hornet (Vespa velutina nigrithorax): The first step toward a green control method
Source: PLoS One. 2020 Oct 6;15(10):e0239742. doi: 10.1371/journal.pone.0239742 (PMC7537856; doi:10.1371/journal.pone.0239742)
Supplement: S1 Table — (PDF) [file pone.0239742.s001.pdf]

Date collection	City	X	Y
07/11/2019	Amboise	47.417943	0.997231
10/07/2019	Amboise	47.408105	0.989489
07/18/19	Artannes-sur-Indre	47.285848	0.590624
07/15/19	Avoine	47.234362	0.220764
05/31/19	Benais	47.298168	0.209658
08/01/2019	Berthenay	47.359124	0.526023
09/03/2019	Bléré	47.321762	0.974438
11/13/19	Bléré	47.324506	1.011413
06/24/19	Bréhémont	47.292395	0.364753
07/15/19	Bréhémont	47.301287	0.371377
11/14/19	Cangey	47.466742	1.056217
07/17/19	Charentilly	47.46973	0.616391
09/10/2019	Chedigny	47.210734	1.006095
10/08/2019	Chedigny	47.20162	1.000347
10/30/19	Chouzée-sur-Loire	47.230105	0.087125
06/03/2019	Civray-de-Touraine	47.337471	1.030889
07/08/2019	Civray-de-Touraine	47.331884	1.057683
07/09/2019	Esvres-sur-Indre	47.3017774	0.800624
07/18/19	Esvres-sur-Indre	47.306735	0.786953
08/13/19	Ferrière-sur-Beaulieu	47.124307	1.067792
07/04/2019	Fondettes	47.407512	0.608172
08/14/19	Fondettes	47.398546	0.631436
07/01/2019	Francueil	47.314288	1.083042
06/12/2019	Ingrandes-de-Touraine	47.286166	0.261942
06/03/2019	Joué-les-Tours	47.350013	0.660949
06/24/19	La chapelle-sur-Loire	47.249413	0.169392
07/15/19	La chapelle-sur-Loire	47.256151	0.227022
06/28/19	La-Ville-aux-Dames	47.399238	0.775097
06/03/2019	Larcay	47.362527	0.774249
05/31/19	Lémeré	47.102791	0.313852
11/15/19	Lignières-de-Touraine	47.289746	0.401646
08/02/2019	Loches	47.094468	0.944278
10/16/19	Loches	47.140533	1.007242
10/17/19	Loches	47.130626	0.997867
05/28/19	Mettray	47.446638	0.655432
07/17/19	Mettray	47.451111	0.642791
06/05/2019	Monnaie	47.511497	0.796626
06/28/19	Monnaie	47.500722	0.79162
06/11/2019	Montlouis-sur-Loire	47.38815	0.821675
08/09/2019	Montlouis-sur-Loire	47.382445	0.813325
09/03/2019	Montlouis-sur-Loire	47.365238	0.862435
11/04/2019	Montlouis-sur-Loire	47.393567	0.850562
07/23/19	Montreuil-sur-Loire	47.488078	0.94417
07/31/19	Parçay-Meslay	47.459969	0.767424
08/09/2019	Parçay-Meslay	47.43919	0.750537
10/01/2019	Pont-de-Ruan	47.26071	0.576437
08/12/2019	Restigne (Fougerolles)	47.283045	0.207188
07/04/2019	Reugny	47.475135	0.880761
05/29/19	Saint-Avertin	47.360988	0.722448

05/06/2019	Saint-Cyr	47.409133	0.677869
06/04/2019	Saint-Cyr	47.413744	0.667257
09/09/2019	Saint-Epain	47.146778	0.516361
07/29/19	Saint-Etienne-de-Chigny	47.368944	0.506104
11/07/2019	Saint-Etienne-de-Chigny	47.400475	0.52421
09/04/2019	Saint-Patrice-sur-Loire	47.275	0.305593
06/03/2019	Saint-Pierre-des-Corps	47.389638	0.710562
06/18/19	Saint-Pierre-des-Corps	47.391269	0.727073
05/29/19	Semblancay	47.505138	0.587109
06/14/19	Tours	47.376591	0.685557
07/10/2019	Tours	47.355971	0.74923
07/19/19	Tours	47.357396	0.7033
05/20/19	Tours_Nord	47.405124	0.685335
06/14/19	Tours_Nord	47.433834	0.702006
09/24/19	Truyes	47.275846	0.827475
09/17/19	Vernou-sur-Brenne	47.415754	0.857849
07/23/19	Vouvray	47.422187	0.799708
