# Supplementary material for: Characterizing thermal tolerance in the invasive yellow-legged hornet (Vespa velutina nigrithorax): The first step toward a green control method
Source: PLoS One. 2020 Oct 6;15(10):e0239742. doi: 10.1371/journal.pone.0239742 (PMC7537856; doi:10.1371/journal.pone.0239742)
Supplement: S2 Table — Normality distribution was tested with Shapiro-Wilk method. Pearson correlation method (with normal distribution) and the Spearman correlation method (without normal disitribution) were used. (PDF) [file pone.0239742.s002.pdf]

Method	Temperature (°C)	Individuals	Test	Correlation coefficient r
Flash	50	Larvae	Pearson	0.165
		Males	Spearman	0.301
		Workers	Spearman	0.606
		Gynes	Pearson	0.287
	60	Larvae	Pearson	0.284
		Males	Pearson	0.332
		Workers	Pearson	0.347
		Gynes	Spearman	-0.153
	70	Larvae	Pearson	0.545
		Males	Pearson	0.606
		Workers	Pearson	0.127
		Gynes	Spearman	-0.075
	80	Larvae	Pearson	0.688
		Males	Pearson	-0.608
		Workers	Spearman	0.571
		Gynes	Spearman	-0.558
Gradual	50	Larvae	Pearson	0.409
		Males	Spearman	0.156
		Workers	Spearman	-0.427
		Gynes	Spearman	-0.034
	60	Larvae	Pearson	0.231
		Males	Pearson	0.151
		Workers	Spearman	-0.445
		Gynes	Spearman	0.056
	70	Larvae	Pearson	0.592
		Males	Pearson	0.159
		Workers	Spearman	-0.291
		Gynes	Spearman	0.0198
	80	Larvae	Pearson	0.609
		Males	Pearson	0.405
		Workers	Spearman	-0.296
		Gynes	Spearman	-0.35
Dry heat	100	Workers	Pearson	0.225
	120	Workers	Spearman	0.162
	140	Workers	Spearman	-0.571
Vapor	92	Gynes	Spearman	0.09
		Males	Spearman	0.289
		Workers	Pearson	-0.08

P-value
0.38
0.106
0.003
0.125
0.124
0.07
0.06
0.415
0.001
0.001
0.504
0.691
0.001
0.723
0.001
0.767
0.249
0.502
0.019
0.853
0.219
0.425
0.137
0.7
0.006
0.403
0.47
0.196
0.003
0.701
0.111
0.057
0.628
0.66
0.15
0.628
0.12
0.671
