# Supplementary figures and images for: Characterizing thermal tolerance in the invasive yellow-legged hornet (Vespa velutina nigrithorax): The first step toward a green control method
Source: PLoS One. 2020 Oct 6;15(10):e0239742. doi: 10.1371/journal.pone.0239742 (PMC7537856; doi:10.1371/journal.pone.0239742)

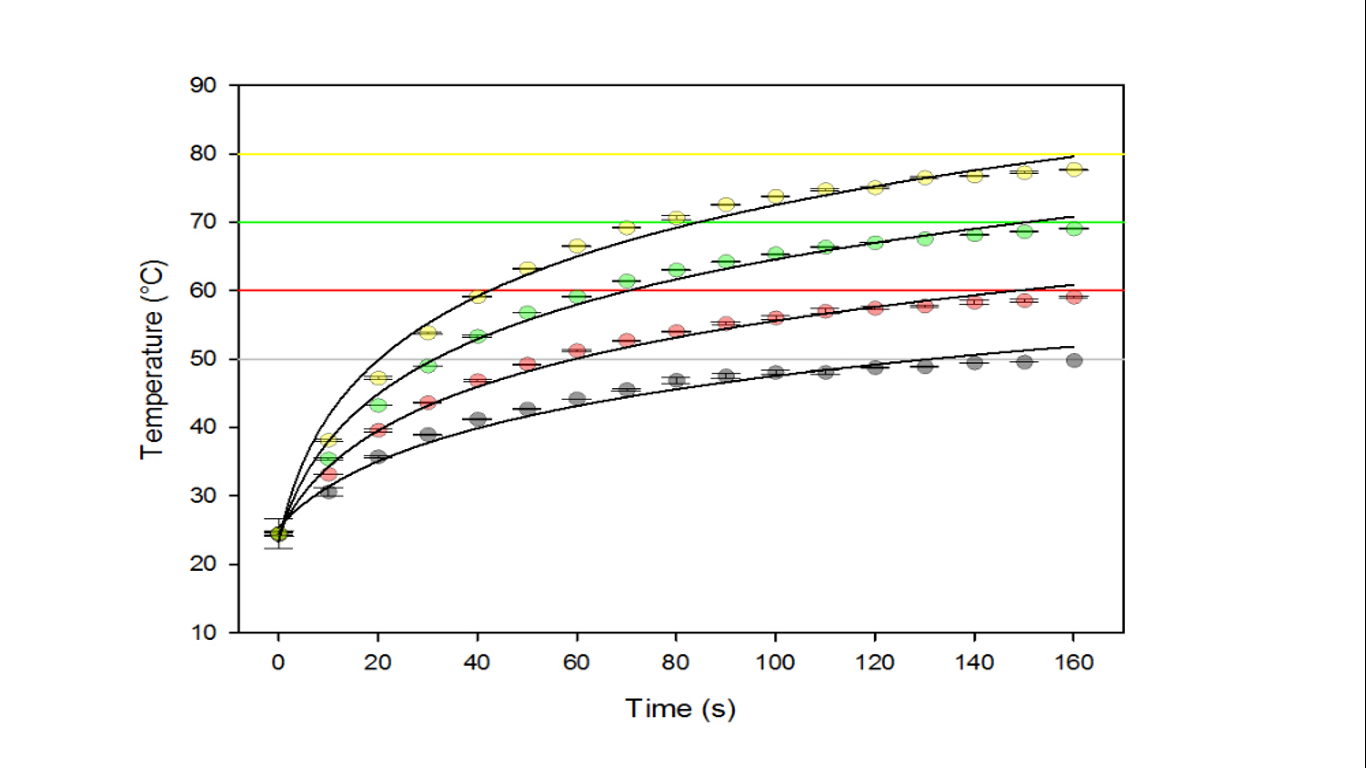

Supplement: S1 Fig — Color codes: gray = 50°C (R2 = 0.9), red = 60°C (R2 = 0.9), green = 70°C (R2 = 0.9), and yellow = 80°C (R2 = 0.9) (n = 3 for each). (TIF) [file pone.0239742.s003.tif]
